# Animal Welfare Monitor: Raising the Bar for Species-Specific Welfare Evaluation Using Welfare Quality^®^ Principles

**DOI:** 10.3390/ani16050842

**Published:** 2026-03-07

**Authors:** Amélie Romain, Léa Briard, Gwenaël Leroutier, Marine Parker, Baptiste Chenet, Constance Wagner, Alexandre Petry, Benoît Quintard

**Affiliations:** 1 Bureau d’études Akongo, 6 rue Suzanne Képès, 44200 Nantes, France; lea.briard@akongo.fr (L.B.); gwenael.leroutier@akongo.fr (G.L.); marine.parker@akongo.fr (M.P.); 2 Zoo de Montpellier, 50 Av. Agropolis, 34090 Montpellier, France; baptiste.chenet@montpellier.fr; 3 Parc Zoologique et Botanique de Mulhouse, 111 Av. de la 1ère Division Blindée, 68100 Mulhouse, France; constance.wagner@m2a.fr (C.W.); benoit.quintard@m2a.fr (B.Q.); 4 Parc Animalier et Botanique de Branféré, 56190 Le Guerno, France; alexandre.petry@branfere.com

**Keywords:** zoo animal welfare, species-specific assessment, AWM assessment, animal-based indicators, Welfare Quality^®^ framework, welfare monitoring methodology, Northern ground hornbill, giraffe

## Abstract

Assessing zoo animal welfare can involve generic evaluations or targeted, species-specific protocols. While the latter offer greater precision, their development is often limited by species diversity and the lack of validated indicators. The Animal Welfare Monitor^®^ (AWM) protocol addresses these challenges by adapting Welfare Quality^®^ principles to zoological contexts. Its core innovation is a four-level hierarchical structure (base, order, family, species) linking broad taxonomic knowledge to species-level protocols. This enables tailored questionnaires for each species, including data-deficient taxa, by leveraging information from related groups. Questionnaires, covering housing, nutrition, health, and behaviour, are complemented by behavioural observations. AWM currently covers 87 species (69 mammals, 15 birds, 2 amphibians, 1 reptile) and constitutes a substantial database of species-specific welfare assessment protocols embedded within a single, standardised methodological framework. Between 2021 and 2025, 14 zoos conducted over 1000 assessments and 15,000 behavioural observations, demonstrating the protocol’s feasibility in routine operations. AWM integrates data entry with visual documentation, such as photographs of enclosures or enrichment, which add context, enhance decision-making, and strengthen long-term records. While refinements such as group-level assessment remain, AWM offers a scalable, flexible tool combining scientific rigour with operational applicability, supporting positive welfare outcomes across diverse zoological institutions.

## Simple Summary

Ensuring good welfare for zoo animals requires tools that capture the specific needs of each species. Generic assessments can overlook important details, while species-specific protocols are often difficult to develop, especially for less-studied taxa. The Animal Welfare Monitor^®^ (AWM) addresses this challenge by adapting the Welfare Quality^®^ framework for zoos, using an innovative four-level structure to design tailored questionnaires. Between 2021 and 2025, it has been applied in 14 zoos. The teams have realised over 1000 assessments, including questionnaires and behavioural observations. This approach provides practical, long-term records that guide daily care, support evidence-based decisions, and improve welfare across institutions.

## 1. Introduction

The question of animal welfare arises whenever humans interact with animals, whether they are farm animals, pets, used in scientific research or human assistance (e.g., animal-assisted therapy, working dogs, etc.), or housed in zoos and aquaria, hereafter simplified as “zoos” [1,2]. This growing concern has been driven not only by scientific advancements but also by their incorporation into regulatory frameworks [3]. Over the past decade, publications on zoo animal welfare have more than doubled [4], underscoring its increasing relevance. In zoos, animal welfare is closely linked to conservation, research, and education, and effective conservation strategies require demonstrable welfare standards [5]. This importance is highlighted by the World Association of Zoos and Aquariums (WAZA) “Caring for Wildlife” global strategy, which emphasises that zoological institutions have an ethical responsibility to ensure the optimal welfare of the animals under their care [6]. As public awareness grows, zoos face increasing expectations to be transparent regarding animal welfare outcomes [7].

Historically, definitions of animal welfare focused on the absence of suffering [1,8]. Contemporary perspectives have, however, expanded this view to include the promotion of positive experiences, such as the expression of natural behaviours and the demonstration of positive emotions [1,9,10,11]. Rault et al. recently established a consensus defining positive animal welfare as “animals flourishing through the experience of primarily positive mental states and the development of skills and resilience,” specifying that this concept goes beyond the mere prevention of suffering [1]. Welfare state is also dynamic, rather than static. It may vary over time and between individuals, even within the same environment [12,13]. Ongoing assessments are therefore essential to capture these fluctuations. Hill & Broom (2009) recommend using individuals as their own controls to track responses to environmental changes better [14]. This variability underscores the need for a multidisciplinary approach that integrates multiple measures to achieve a holistic understanding of animal welfare [15].

Today, animal welfare is recognised as a multidimensional concept encompassing behavioural, emotional, and physiological states, shaped by an animal’s environment and life history [8,16,17]. Developed in 1994 by Mellor & Beausoleil, the Five Domains Model provides a comprehensive framework by distinguishing four physical domains—nutrition, environment, health, and behaviour (recently redefined as “behavioural interaction”)—and a fifth domain representing the animal’s mental state [18]. Although the model continues to evolve with new scientific insights, its practical application as a welfare assessment tool remains challenging, as no standardised assessment protocol is directly linked to this framework [19,20].

A significant step towards standardising welfare assessment methods was the Welfare Quality^®^ (WQ) project [21,22]. This European initiative aimed to harmonise assessments of farm animals in different farm settings and countries. Although the Welfare Quality^®^ protocols are conceptually consistent with the Five Domains framework, they were primarily developed as operational assessment tools. Welfare Quality^®^ protocols are species-specific (e.g., cattle, chickens, and pigs). Rather than directly evaluating mental states, these protocols infer welfare outcomes through the integration of resource-based, management-based, and animal-based indicators [21,23]. Management-based measures (e.g., food distribution timing) have also been recognised for their role in welfare, as daily practices can significantly affect it [24,25].

In zoo contexts, a wide range of welfare assessment approaches has been explored. Tallo-Parra et al. (2023) identified several types, including species-specific assessments (often adaptations of Welfare Quality^®^), generic protocols developed by both researchers and professional organisations (e.g., European or National associations), time budgets, keeper ratings (such as the Qualitative Behavioural Assessment—QBA) and cognitive bias tests [26]. Each method has distinct strengths and limitations. Generic protocols, for example, can help identify welfare risks and prioritise improvements, but they often lack the adaptability required for the diversity of zoo species. They may rely on subjective evaluations and variable levels of user knowledge regarding species biology and needs, and they can struggle to balance the multidimensional aspects of welfare [27,28]. Keeper ratings can be reliable and routine-integrated but might be influenced by an emotional attachment to the animals under their care [27,29]. Cognitive bias tests, though valuable for assessing emotional valence, remain time-consuming and under-researched [26].

In response to these challenges, many approaches have evolved towards questionnaire-based assessment systems, sometimes adapted from protocols originally designed in laboratory or farm settings. For instance, the Animal Welfare Assessment Grid (AWAG), originally designed for laboratory animals (e.g., primates, dogs), has been adapted and tested in South Korean zoos [30]. Racciatti et al. proposed the Ackonc-AWA protocol [25], which integrates questionnaires with behavioural observations to facilitate team involvement. Other adaptations include the Simplified Animal Welfare Assessment Grid (S-AWAG) designed for smaller zoos [31] and species-specific validations of AWAG, such as for gorillas (*Gorilla gorilla gorilla*) through keeper ratings [32]. Although these generic tools are flexible across species, they often place limited emphasis on species-specific needs [33] and require users to have substantial knowledge of species-specific biology, recommendations, or legislation [28].

Species-specific assessments tackle these limits. Examples include the C-Well^®^ protocol for bottlenose dolphins (*Tursiops truncatus* [34]), which was later expanded into Dolphin-WET [14] and, more recently, adapted to five cetacean species [35]. Additional examples include adaptations of the Welfare Quality^®^ protocol for dorcas gazelles (*Gazella dorcas* [36]), elephants *(Loxodonta africana* and *Elephas maximus* [37]) and the pygmy blue-tongue skink (*Tiliqua adelaidensis* [38]). These studies highlight the value of comprehensive, standardised frameworks for wild species. Such frameworks integrate multiple welfare dimensions and promote ethical practices by focusing on animal-based indicators and overall quality of life [26,36,39,40].

There is therefore a growing demand for species-specific, standardised, and adaptable welfare assessment tools [27,33]. Their implementation in zoos nevertheless remains challenging, as each species requires a tailored protocol that accounts for its unique biological, behavioural, and environmental needs. Persistent limitations include a strong focus on flagship species, limited resources for less-studied taxa (e.g., small mammals, birds, amphibians, reptiles), difficulties in monitoring welfare across entire lifespans, and considerable heterogeneity in both the content and application of existing protocols [4,26]. Additionally, domestic species housed in petting zoos are often overlooked, despite fitting neatly into neither the farm nor the exotic animal categories.

In response to these limitations, the Animal Welfare Monitor (AWM) protocol has been developed. Firmly grounded in Welfare Quality^®^ principles and designed to address the shortcomings of previous approaches, AWM was created in collaboration with animal care teams. Its aim is to provide a species-specific tool that offers tailored recommendations and includes underrepresented species. By combining questionnaires with direct behavioural observations, the AWM method captures both positive indicators (e.g., affiliative behaviours, optimal body condition, species-adapted food presentation) and negative indicators (e.g., abnormal behaviours, excessive agonistic behaviours, inadequate housing). This approach enables a comprehensive and accurate assessment of animal welfare [16,22,41].

The primary objective of the AWM protocol is to provide a user-friendly, species-specific welfare-monitoring tool that enables zoo teams to track changes in welfare over time. This study describes the development and key methodological principles of the AWM framework, presents its main features, and examines its practical utility, strengths, and limitations in zoological settings. The implementation and adaptability of the methodology are illustrated through two demonstration case studies involving giraffes (*Giraffa camelopardalis*) and Northern ground hornbills (*Bucorvus abyssinicus*), and future research directions are outlined.

## 2. Materials and Methods

### 2.1. Overview of the Animal Welfare Monitor (AWM) Framework

The Animal Welfare Monitor (AWM) was developed as a species-specific welfare assessment tool tailored to the ecological diversity and operational constraints of zoological institutions. Inspired by the Welfare Quality^®^ framework, AWM addresses the limitations of existing approaches, which are often either overly generic or restricted to a limited number of well-studied species.

A core objective of AWM was to design a practical, accessible tool that could be used reliably by all zoo professionals, including keepers, veterinarians, and curators, regardless of prior expertise in animal welfare science. The protocol is structured to guide users step by step through the assessment process, combining questionnaire-based indicators with structured behavioural observations.

**Figure 1:**
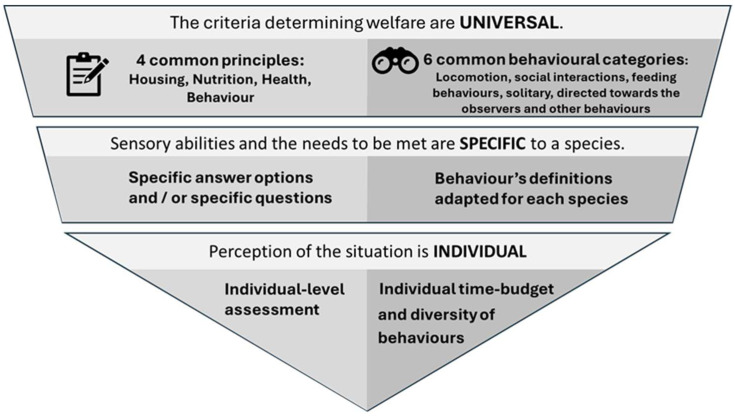
Overview of the AWM framework: universal welfare principles, species-specific questionnaire adaptation, and individual-level assessment.

As illustrated in Figure 1, the AWM framework is organised across three complementary levels. First, welfare is assessed using universal criteria derived from four overarching principles—housing, nutrition, health, and behaviour—together with six standardised behavioural categories applicable across taxa. Second, these criteria are refined at the species-specific level through tailored questions, response options, and behaviour definitions adapted to the biological and ecological characteristics of each taxon. Finally, welfare is assessed at the individual level, integrating behavioural diversity, time budgets, and the animal’s observed responses to its environment.

By linking environmental and management inputs with animal-based behavioural outputs, this multi-level structure allows the identification of both potential risk factors and their observable consequences. The AWM protocol relies exclusively on non-invasive indicators and was designed to be resource-efficient, thereby facilitating its implementation across a wide range of zoological contexts. The protocol is designed to support iterative welfare management.

### 2.2. Questionnaire

#### 2.2.1. Principles, Criteria and Indicators

The development of the AWM questionnaire followed a two-step process. First, a critical review of the Welfare Quality^®^ principles, criteria, and indicators was conducted to evaluate their applicability to zoological settings. This preliminary analysis was complemented by case studies (e.g., giraffes) to identify indicators that could be directly transferred, adapted, or considered unsuitable for zoo environments. Based on this assessment, the four overarching Welfare Quality^®^ principles—Good Feeding, Good Housing, Good Health, and Appropriate Behaviour—were retained as the conceptual foundation of the AWM protocol. These principles address both physical and psychological dimensions of welfare, encompassing basic biological needs as well as the expression of species-typical behaviours.

In a second step, the original Welfare Quality^®^ criteria were adapted or redefined to reflect better the diversity of species, enclosures, and management practices encountered in zoological institutions. Zoo-specific considerations, such as enclosure access, training routines, and behavioural monitoring practices, were explicitly integrated. This process resulted in 20 criteria: nine related to housing, three to nutrition, three to health, and five to behaviour.

The final set of criteria (Table 1) provides an operational framework for welfare assessment in zoos while maintaining conceptual continuity with the Welfare Quality^®^ model. The measures are animal-based, resource-based, and management-based indicators. The repartition of the measures is detailed in Table S1. Animal-based measures serve as outputs, while resource- and management-based indicators represent environmental inputs. While management- and resource-based indicators do not directly measure affective states, they represent actionable leverage points for welfare improvement. They are systematically linked to behavioural outputs within the AWM framework. This structure facilitates data collection and interpretation across taxa and enables intuitive visualisation of results, particularly through radar charts.

#### 2.2.2. Hierarchical Structure (Base, Order, Family, and Species)

To accommodate the biological and ecological diversity of zoo-housed species while maintaining consistency across assessments, the AWM questionnaire is structured according to a four-level hierarchical system: base, order, family, and species.

The base level includes generic indicators that can be assessed across all taxa, such as enclosure characteristics or general clinical signs. These base questions provide a common reference framework and ensure comparability across species. When necessary, successive variant levels are introduced to refine the assessment and improve taxonomic relevance.

Adaptations at the order, family, or species level involve one or more of the following modifications: (i) adjustments to question wording to reflect anatomical or physiological differences (e.g., skin, feathers, or scales), (ii) modification of response options to include taxon-specific signs or behaviours, and (iii) adjustment of weighting coefficients to reflect the relative welfare relevance of specific indicators within a given taxon.

**Figure 2:**
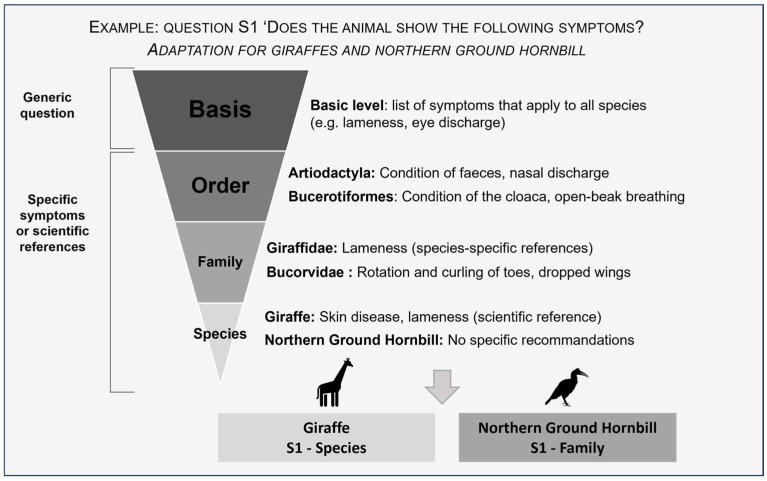
Procedure for adapting questions across taxonomic levels (base, order, family, species), illustrated with a health-related indicator for giraffes and Northern ground hornbills.

For example, a health-related question assessing clinical symptoms is defined at the base level using general signs observable across species. It is progressively refined for specific orders, families, and species by integrating additional, taxon-relevant clinical indicators supported by the literature (Figure 2).

This hierarchical structure enables consistent assessment of the same individual across different life stages and management contexts, while ensuring that the questionnaire remains adaptable and can be updated as new scientific evidence emerges.

#### 2.2.3. Species-Specific Adaptation and Sources of Information

The AWM protocol was designed to apply to all species housed in zoological institutions, including both wild and domesticated taxa. For each species, the questionnaire’s development integrates multiple sources of information, including peer-reviewed scientific literature, husbandry guidelines issued by professional organisations (e.g., European Association of Zoos and Aquaria, Association of Zoos & Aquariums, Pan-African Association of Zoos & Aquaria, Global Federation of Animal Sanctuaries), and relevant national or international legislation.

Given that published husbandry guidelines are not always regularly updated, this evidence-based approach was complemented by grey literature, expert consultation, and practical input from zoo professionals. Management-based expertise was significant for indicators related to enrichment quality, feeding strategies, and daily husbandry practices, recognising that long-term experience can provide valuable welfare-relevant insights.

For domesticated species commonly housed in zoos (e.g., donkeys, goats, rabbits), findings from farm and laboratory studies were also incorporated when relevant, thereby integrating established knowledge on species-specific needs and training responses.

The development of species-specific questionnaires for giraffes (*Giraffa camelopardalis*) and Northern ground hornbills (*Bucorvus abyssinicus*) illustrates contrasting data availability. Giraffes benefit from extensive behavioural and husbandry literature, which facilitates the development of detailed welfare indicators [42,43,44,45,46]. In contrast, Northern ground hornbills (NGH) are underrepresented in the scientific literature, requiring reliance on broader taxonomic references and limited species-specific studies. Despite being of conservation concerns, NGH are significantly underrepresented in the formal scientific literature. Information on their behavioural needs, social complexity, and cognitive abilities remains sparse, and existing husbandry guidelines are either limited or generalised at the family or order level [47]. These examples highlight the need for flexible, multi-source methodologies when designing welfare assessment tools for taxonomically diverse zoo species.

#### 2.2.4. Scoring System and Weighting Procedure

The AWM protocol uses a structured scoring system to generate quantitative welfare scores for each of the four main principles: housing, nutrition, health, and behaviour. To prevent compensation effects, scores obtained for one principle cannot offset deficiencies in another, ensuring that critical welfare issues are not masked by high performance in unrelated domains.

Each indicator is assessed through one or more questions with predefined response options, scored on a standardised five-level scale ranging from −1 (negative impact on welfare) to +3 (strongly positive contribution to welfare). This scale is applied consistently across animal-based, resource-based, and management-based indicators to ensure comparability between assessments (Table 2).

Each question is assigned a weight based on the parameter importance for animal welfare. While most questions are assigned a standard coefficient of 1, some indicators are weighted differently to reflect their relative importance in assessing the animal’s welfare (examples are presented in Table S2—see Supplementary Materials). Weighting coefficients were defined based on ecological relevance and expert consensus involving animal care professionals and welfare scientists. Proposed weighting coefficients were discussed collectively during development workshops and refined through repeated application in operational settings. In addition, we conducted a sensitivity analysis using empirical data (34 questionnaires from three giraffes) to test alternative weighting scenarios (equal weights and increased weights). Results showed only small and predictable changes in scores, without altering the overall welfare interpretation across individuals. These results are presented in Table S3. Weighting therefore affects score magnitude but does not alter the qualitative welfare profile, and coefficients can be updated as new evidence becomes available.

Therefore, the contribution of each indicator to the overall welfare score of its category is modulated by two distinct parameters: the score (S) associated with each response option (Table 2) and the weight (W) assigned to the indicator. This dual mechanism ensures that indicators with greater relevance—such as those related to, for instance, social housing, clinical symptoms, or abnormal behaviour—have a proportionally greater impact on the global welfare assessment. It thereby avoids uniform weighting across indicators and supports a more sensitive and context-relevant evaluation. For specific multiple-choice questions, maximum scores are capped to prevent penalising institutions when multiple appropriate options exist, but not all are simultaneously applicable. This approach ensures that scoring reflects welfare quality rather than checklist completion.

We compute an adjusted score (${AS}_{i}$) for each indicator as:

$${AS}_{i}=W_{i}\times S_{i}$$

**Figure 3:**
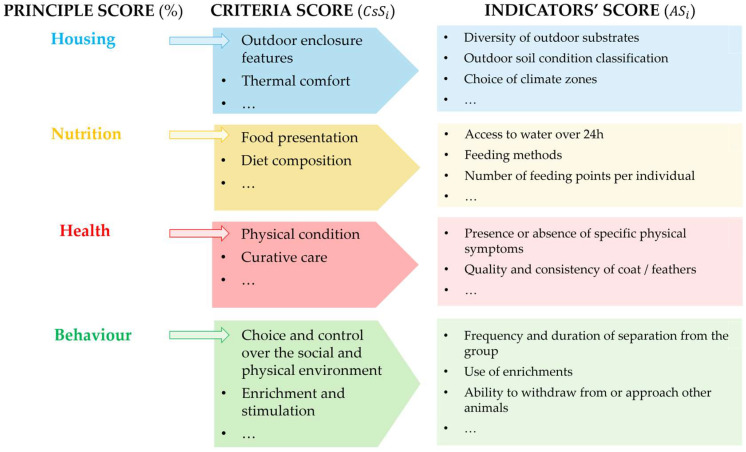
Model for the calculation and aggregation of welfare scores at indicator, criterion, and principle levels. The diagram shows how individual answers are weighted to produce aggregate scores at the criteria and principle levels.

Criterion scores are then obtained by aggregating adjusted indicator scores and expressed as percentages (0–100). Principle scores are calculated by aggregating criterion scores within each principle (Housing, Nutrition, Health, Behaviour). **Full formulas and details are provided in Supplementary Methods S1.** The full calculation workflow is illustrated in Figure 3.

For each criterion, indicator scores are aggregated bottom-up to produce criterion-level scores, which are then integrated into principle-level scores. Final principle-level scores are expressed as percentages to facilitate interpretation, visualisation, and longitudinal comparison across assessments (Figure 3).

The primary purpose of the scoring system is to monitor changes in welfare over time rather than to define absolute thresholds. The AWM protocol therefore specifically targets the areas that require attention and recognises that establishing absolute reference values is challenging. Each animal therefore serves as its own reference point, allowing the detection of meaningful trends in welfare in relation to environmental, management, or social changes.

#### 2.2.5. Use of Visual Media

To promote consistent interpretation of indicators and reduce observer-related variability, the AWM questionnaire integrates visual reference materials throughout the assessment process. These resources include photographs and, where relevant, short video sequences illustrating key animal-based indicators such as body condition, integument condition (fur, feathers, scales, skin), mouthparts, and limb or foot condition.

Inspired by the approach used in Welfare Quality^®^ protocols, these visual aids serve as practical scoring references rather than evaluative tools. They are designed to support users with different levels of experience and to enhance the reproducibility of assessments across institutions and observers.

In addition to reference materials embedded in the questionnaire, photographic and video documentation of enclosures, feeding areas, and enrichment devices can be collected during assessments. Although these visual records are not scored and do not directly influence the overall evaluation outcome, they provide valuable contextual information, can facilitate internal communication and provide valuable reference points over time to promote qualitative long-term monitoring.

### 2.3. Behavioural Observations

Behaviour provides direct insight into how animals perceive and interact with their environment and therefore represents a key component of animal welfare assessment [41,48]. Behavioural observations complement questionnaire-based indicators by capturing welfare-relevant information that may not be detectable through resource- or management-based measures alone [22]. This approach aligns with current recommendations advocating multidimensional welfare assessment frameworks that integrate both positive (e.g., affiliative behaviours, play) and negative (e.g., stereotypies, self-directed abnormal behaviours) indicators [48]. Behavioural monitoring also facilitates the identification of environmental or management-related constraints underlying welfare issues, supporting targeted corrective actions.

#### 2.3.1. Behavioural Categories and Ethogram Design

The AWM protocol integrates standardised, species-specific ethograms to support quantitative behavioural assessment across taxa. Behavioural observations are structured around six core categories applicable to all species: exploration and locomotion, social behaviours, feeding behaviours, individual behaviours, behaviours directed towards the observer, and other behaviours.

Each category provides complementary insights into welfare: exploratory and foraging behaviours encompass actions through which animals interact with objects or environments; they serve as proxies for cognitive engagement and environmental curiosity and are often reflective of positive welfare states. They also encompass repetitive behaviours, such as pacing. Social behaviours, meanwhile, provide insight into group dynamics, hierarchies, benefits and potential stressors arising from social interactions. Feeding behaviours reveal the integrity of natural feeding sequences and possible issues related to dietary management. Individual behaviours, including self-directed actions such as grooming or resting, offer additional indicators of the animal’s internal state. In contrast, behaviours directed towards the observer elucidate the influence of human presence on the animal, an aspect critical in zoo settings. The “other” category captures unclassified behaviours and visibility gaps.

Species-specific ethograms were developed by adapting a common behavioural framework using systematic reviews of ethological and welfare literature. Existing ethograms were synthesised and refined to reflect each species’ natural behavioural repertoire and ecological context [49,50].

Examples of ethogram adaptations are provided in Table 3, with full ethograms available in the Supplementary Materials (Table S4).

Key behaviours like exploration, foraging, and social interaction are central to welfare but must be contextualised within species-specific ecological and behavioural repertoires.

#### 2.3.2. Observation Method: Focal Animal Sampling

Behavioural data were collected using focal animal sampling [51], a widely used method in ethology that allows detailed and reliable recording of individual behaviours. Each focal animal was observed for predefined time intervals, during which all behaviours were recorded continuously using the species-specific ethogram. Focal sampling was selected for its ability to accurately estimate the frequency and duration of behaviours at the individual level, including rare or short-duration events. This method supports the assessment of behavioural diversity and enables the detection of both positive and negative welfare-related behaviours.

#### 2.3.3. Observer Training and Bias Minimisation

To ensure data quality and consistency, observers received standardised training prior to data collection, including familiarisation with the species-specific ethogram and scoring procedures. Training sessions were aimed at harmonising behavioural definitions and minimising interpretative variability among observers.

To minimise inter-observer variability, animal care staff followed a standardised training programme in dyads based on focal sampling. Training sessions were conducted on the same individuals, followed by structured debriefings to harmonise the ethogram and decision rules. Our team supported calibration throughout by clarifying the ethogram and its operational definitions (including adding precision when needed) and, when possible, providing short video excerpts illustrating key behaviours and common sources of ambiguity.

Inter-observer reliability was assessed on paired sessions using percent agreement, Cohen’s κ (second-by-second agreement), and an intraclass correlation coefficient (ICC) on 30 s windows (agreement on time spent per behaviour). Dyads were considered validated when κ ≥ 0.61 and ICC ≥ 0.75. When thresholds were not met, observers received targeted feedback and the test was repeated. Full reliability procedures, interpretation thresholds, and the networked calibration approach are described in Supplementary Methods S2.

Because observations were conducted live (as per the intended field use), sequences could not be replayed for post hoc adjudication, which may limit standardization compared with video-based coding; however, reliability was quantified under operational conditions using paired sessions and inter-rater metrics (percent agreement, Cohen’s κ, and ICC), with iterative calibration and re-testing when thresholds were not met. When disagreements involved rare behaviours (e.g., social interactions) or ambiguous cases, observers discussed the episode after the session, re-checked the ethogram definitions, and consulted our team as needed before repeating the training/test session. For larger teams and/or operational constraints where not all potential observer pairings could be tested directly (e.g., staff with limited overlap), we used a networked calibration approach whereby each observer was required to meet the above reliability thresholds with at least one trained “anchor” observer who had also demonstrated agreement with other team members, ensuring consistency across the group despite incomplete pairwise training. An example of inter-observer reliability results is presented in Table S5 (Supplementary Materials).

During observations, potential observer effects were controlled by implementing a brief habituation period before data collection and by systematically recording behaviours directed toward the observer. Environmental or contextual disturbances occurring during observation sessions were documented to support interpretation of the data.

#### 2.3.4. Behavioural Data Analysis and Time-Budget Construction

The observational method leverages time-budget analyses to deliver a comprehensive, dynamic evaluation of zoo animal welfare, supporting both immediate assessments and long-term monitoring.

Behavioural observations were analysed using time-budget approaches to quantify the proportion of time allocated to different behavioural categories. This analytical framework allows the evaluation of whether an individual’s behavioural repertoire aligns with patterns associated with welfare states. For data analysis, behaviours recorded during observations were grouped into analytical categories reflecting their welfare relevance. For analysis, recorded behaviours were regrouped into welfare-relevant analytical categories (Table 4). This analytical grouping differs from the observation ethogram structure, which is designed to reduce observer bias during recording. For instance, behaviours with similar welfare implications were pooled under abnormal behaviours (e.g., pacing, repetitive oral behaviours).

Time-budget analyses support both cross-sectional welfare assessments and longitudinal monitoring by enabling comparisons within individuals over time and with species-specific reference patterns when available. As highlighted by Salas et al. (2024), exploring individual variations provides a nuanced view of welfare, allowing for comparisons between an individual’s current state and species-specific benchmarks derived from natural settings, as well as with its own historical data [48].

### 2.4. Data Collection Strategy and Assessment Frequency

#### 2.4.1. Assessment Frequency and Longitudinal Monitoring

The Animal Welfare Monitor (AWM) protocol is designed to support repeated welfare assessments throughout an animal’s lifespan, with particular attention to periods of significant change, such as modifications in housing, management practices, group composition, or health status. To account for seasonal variation and changes in enclosure use, questionnaire-based assessments are recommended to be conducted two to four times per year. These repeated assessments allow monitoring of environmental conditions, husbandry practices, and welfare-related outcomes over time. When animals are housed under similar conditions, some questionnaire items may be assessed at the group level to improve feasibility (e.g., the questionnaire can typically be completed in approximately 45 min for the first animal and 10 min for each additional individual).

A comprehensive welfare assessment combining questionnaire data and behavioural observations should be conducted at least twice per year to provide a robust baseline and support longitudinal welfare monitoring.

#### 2.4.2. Behavioural Observation Scheduling

Behavioural data collection is conducted over defined observation periods to allow the construction of individual time budgets. The AWM protocol prioritises focal continuous sampling, which provides detailed information on behavioural frequency, duration, and sequencing [52,53]. This approach also accounts for intra-individual variability, supporting a more nuanced and personalised welfare assessment [54].

Importantly, recent findings by Wilder et al. highlight that the validity of behavioural data depends more on total observation time than on the specific sampling method [55]. Accordingly, the AWM protocol emphasises cumulative observation effort distributed across different times of day rather than fixed or convenience-based sampling windows.

#### 2.4.3. Feasibility and Operational Constraints

Given that the AWM protocol is intended for routine use by animal care teams, operational feasibility was a key design consideration. Care staff often work under time constraints and may not be supported by external researchers.

To accommodate these realities, the protocol recommends relatively short focal observation sessions (approximately 10 min), repeated across multiple days and time points to maximise representativeness while limiting observer burden [37,56,57]. Observation sessions are distributed across the facility’s active hours using a pseudo-randomised schedule, ensuring a balance between morning and afternoon periods and reducing potential order effects. This planning might also be adapted to nocturnal activities, for example, by using cameras, to support a 24/7 approach [58].

The use of digital data collection tools, such as mobile applications, is encouraged to facilitate behavioural recording, reduce transcription errors, and support longitudinal data management (e.g., the Akongo mobile app, *Nantes*, *France*—as well as other tools; for a review, see Van der Marel et al., 2022 [59]).

Observation frequency and intensity should remain flexible and be adjusted according to institutional capacity, particularly following significant changes in animal management or social structure.

## 3. Results

Together, questionnaire-based assessments and behavioural observations generated complementary outputs that informed both welfare evaluation and management decisions. The following sections present how these outputs were used in practice to (i) identify welfare priorities, (ii) support the interpretation of assessment results, and (iii) assess the feasibility of implementing the AWM protocol across zoological institutions.

### 3.1. Use of AWM Outputs to Inform Welfare Assessment and Management

The Animal Welfare Monitor (AWM) protocol generates integrated outputs from questionnaire-based assessments and behavioural observations, enabling the identification of welfare priorities and the evaluation of management interventions over time. The following case studies are presented as descriptive demonstrations of how complementary AWM data streams can be used in practice to support welfare-related decision-making at individual and group levels. They are intended to illustrate feasibility and applied use, rather than to provide formal validation or causal evaluation of welfare interventions.

#### 3.1.1. Visual Representation of Welfare Assessments

**Figure 4:**
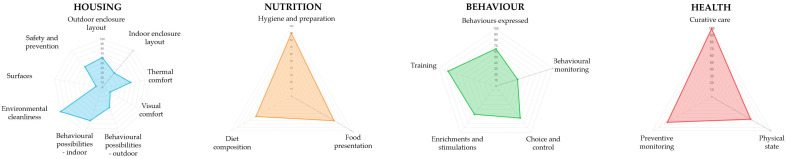
Radar charts displaying criterion-level welfare scores (0–100%) for an adult male giraffe, presented separately for Housing, Nutrition, Health, and Behaviour. Lower-scoring criteria indicate priority areas for improvement; points closer to the chart’s outer edge indicate higher scores.

Questionnaire results were visualised using radar charts, with one chart per welfare principle (Housing, Nutrition, Health, Behaviour) and each axis representing a criterion scored from 0 to 100%. These visualisations allow rapid identification of criteria with lower scores and facilitate comparisons across assessment periods within the same principle (Figure 4).

**Figure 5:**
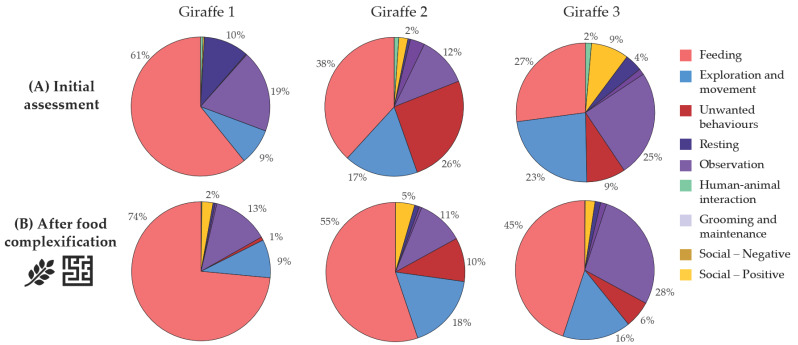
Activity budgets derived from 10 min focal observations for three male giraffes before (**A**) and after (**B**) food presentation complexification. Sample sizes: n = 40 (Giraffe 1), n = 37 (Giraffe 2), n = 36 (Giraffe 3).

Behavioural observation data were summarised as activity budgets, presented as pie charts showing the proportion of time allocated to different behavioural categories (see Figure 5 in the following section). Together, radar charts and activity budgets supported both individual-level assessment and longitudinal monitoring of changes in welfare.

#### 3.1.2. Combined Questionnaire and Behavioural Data to Prioritise Interventions: Giraffe Demonstration Case Study

Initial AWM assessments conducted on a bachelor group of adult male giraffes revealed suboptimal scores in several criteria within the Housing, Nutrition, and Behaviour principles (Figure 4). Enclosure size received the lowest score, although this factor was identified as difficult to modify in the short term. Lower scores were also observed for criteria related to food presentation and environmental enrichment.

Behavioural observations supported these findings. Two individuals (Giraffe 2 and Giraffe 3) showed relatively low proportions of feeding and ruminating behaviours during daytime observations (38% and 27%, respectively), combined with higher proportions of oral stereotypic behaviours such as surface licking (9% and 26%) (Figure 5A). The questionnaire further indicated mismatches between diet characteristics and species-specific nutritional requirements, as well as limited feeding complexity.

**Figure 6:**
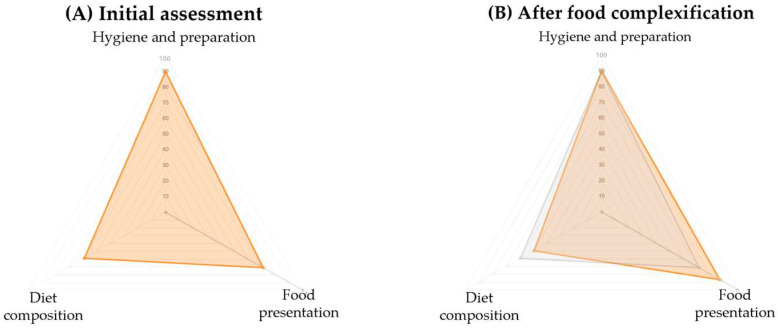
Changes in the “Food presentation” criterion score following dietary enrichment intervention in giraffes.

Following the implementation of targeted measures to increase food presentation complexity—such as the introduction of puzzle feeders, modified hay racks, and additional feeding stations—the score for the “Food presentation” criterion increased from 71% to 90%. The scores for the other nutrition-related criteria remained unchanged (Figure 6). Post-intervention behavioural observations showed an increase in feeding and ruminating behaviours across all individuals (Giraffe 1: 61% to 74%; Giraffe 2: 38% to 55%; Giraffe 3: 27% to 45%) and a reduction in abnormal behaviours for Giraffes 2 and 3 (Figure 5B).

#### 3.1.3. Use of Behavioural Data to Monitor and Adjust Ex Situ Population Management Practices, and Individual Welfare over Time: Northern Ground Hornbill Demonstration Case Study

In a second case study, behavioural monitoring was used to assess changes in welfare for a female Northern ground hornbill (*Bucorvus abyssinicus*) experiencing successive social contexts. The individual was initially housed in a stable social pair with her sister, before undergoing a temporary period of social isolation following her partner’s transfer as part of an ex situ breeding programme. Given the species’ social nature, this new housing condition was recognised as a potential welfare risk, prompting the team to maintain regular behavioural monitoring throughout the transition.

**Figure 7:**
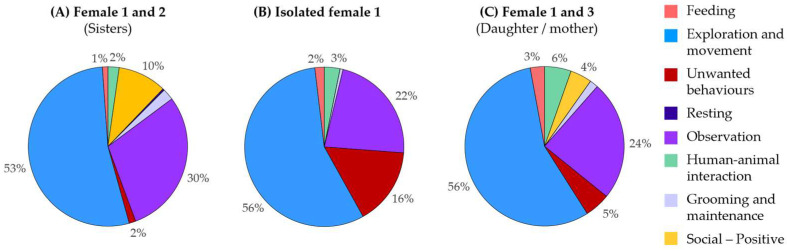
Longitudinal monitoring in a female Northern ground hornbill across three social contexts: (**A**) paired with her sister, (**B**) temporary social isolation, and (**C**) paired with her mother. Outputs are shown for successive assessment periods to illustrate welfare changes over time.

Behavioural observations conducted during the isolation period revealed an increase in indicators associated with reduced welfare, including a rise in stereotypic behaviours (notably pacing, from 2% to 16%) and a slight increase in human-directed behaviours, alongside the absence of social interactions (Figure 7). To mitigate these changes, a temporary social companion (the individual’s mother) was introduced.

Subsequent behavioural monitoring showed a reduction in stereotypic behaviours (from 16% to 5%) and the reappearance of social interactions, although the behavioural profile differed from that observed in the original social pair (Figure 7). These results illustrate the sensitivity of behavioural time budgets to changes in social context and the value of behavioural monitoring for tracking individual welfare during population management transitions.

In addition to numerical scores and behavioural time budgets, supplementary information was used to support the interpretation of welfare assessments, particularly in contexts involving environmental or seasonal variation. The contribution of visual media to welfare assessment interpretation is presented below.

### 3.2. Contribution of Visual Media to Welfare Assessment Interpretation

Although the use of photographs and videos is optional within the AWM protocol, visual media provided valuable complementary information to numerical welfare scores in several assessment contexts. Visual documentation supported the interpretation of questionnaire results by contextualising environmental conditions and facilitating the prioritisation of welfare interventions.

**Figure 8:**
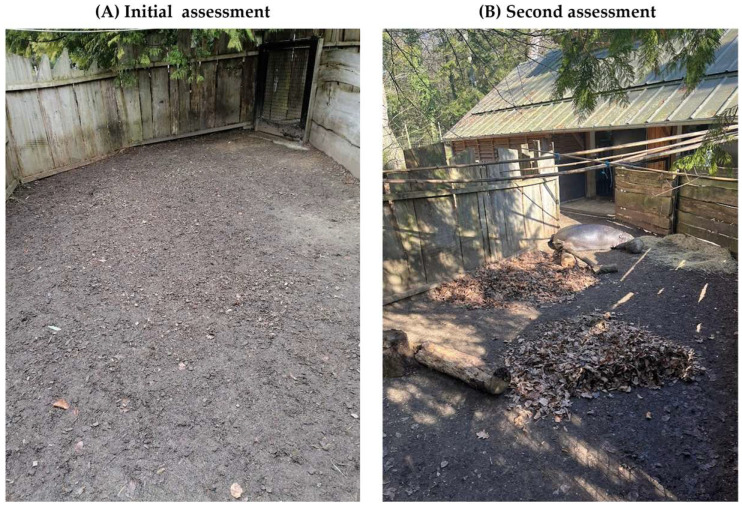
Photographic comparison of a pygmy hippopotamus winter enclosure (**A**) before and (**B**) after structural modifications.

For example, in pygmy hippopotamuses (*Choeropsis liberiensis)*, photographs taken during winter assessments documented the use of a corral area that functioned as the primary outdoor enclosure during that season. At the initial evaluation, the “Enclosure layout” criterion received a low score (29%), and visual documentation highlighted limited substrate diversity and structural complexity. Following targeted modifications, including the addition of varied substrates and structural elements, the score increased to 63%, with photographs providing clear visual evidence of environmental changes (Figure 8).

Visual media also supported longitudinal interpretation of welfare assessments by documenting environmental variation outside direct management control. In a group of greater kudus (*Tragelaphus strepsiceros*), a series of photographs taken across multiple seasons documented changes in vegetation cover, ground conditions, and shade availability, including the visible effects of drought (Figure S1—see Supplementary Materials). These images provided contextual information that complemented questionnaire scores and facilitated the interpretation of welfare trends over time.

Overall, visual media contributed to a more comprehensive understanding of welfare assessments by supporting score interpretation, documenting environmental change, and enhancing longitudinal monitoring, particularly in situations where seasonal or contextual factors influenced enclosure conditions.

Beyond their contribution to the interpretation of individual assessments, the use of the AWM protocol generated a substantial body of implementation data across institutions. The following section presents results on the uptake, taxonomic coverage, and operational feasibility of the protocol in routine zoo settings.

### 3.3. Feasibility and Uptake of the AWM Protocol Across Zoological Institutions

The feasibility of the AWM protocol was evaluated through its implementation across multiple zoological institutions over a four-year period (2021–2025), focusing on taxonomic coverage, questionnaire use, and behavioural observation effort.

#### 3.3.1. Taxonomic Coverage of the AWM Protocol

Since its implementation, the AWM protocol has been applied to 87 species housed in zoological institutions, including 69 mammal species, 15 bird species, two amphibian species, and one reptile species. These species span a wide range of taxonomic orders (Table 5), demonstrating the adaptability of the protocol across diverse taxa, and the full list of species is provided in Table S6 (see Supplementary Materials).

Most species-specific questionnaires currently concern mammals, reflecting the priorities expressed by participating institutions. However, the number of bird species included has increased steadily over time, and preliminary work has begun for reptiles and amphibians, highlighting the protocol’s potential for expansion to underrepresented taxa.

While taxonomic coverage reflects the conceptual adaptability of the AWM framework, questionnaire completion rates provide insight into its practical integration within institutional workflows.

#### 3.3.2. Questionnaire Implementation Across Institutions

Between 2021 and 2025, a total of 1018 questionnaires were completed across 14 zoological institutions, covering both baseline assessments and post-intervention evaluations. The number of species monitored per institution ranged from four to 66, with most institutions assessing between 10 and 21 species over the study period (Table 6).

Quarterly analyses indicated that, in most cases, fewer than 10 species were assessed within the same quarter (see Figure S2 in Supplementary Materials for detailed results), reflecting variations in institutional priorities and available resources. Although the recommended assessment frequency was up to four questionnaires per species per year, this frequency was rarely achieved in practice. Across 174 species monitored in 14 institutions, assessment frequency declined over time, with the majority of species assessed once or twice per year rather than quarterly (Table 7).

#### 3.3.3. Behavioural Observation Effort and Participation

To assess the protocol’s integration into routine zoo operations, the primary analysis focuses on the 15,934 behavioural observation sessions (10 min each) conducted exclusively by in-house staff. When including additional support from external researchers and interns, the total volume of data collected reaches 23,926 sessions. A comparison of the number of observations between in-house staff and all participants is presented in Figure S3 (see Supplementary Materials). In total, 295 users contributed to at least one observation session during the four-year period, reflecting broad staff participation across institutions.

**Figure 9:**
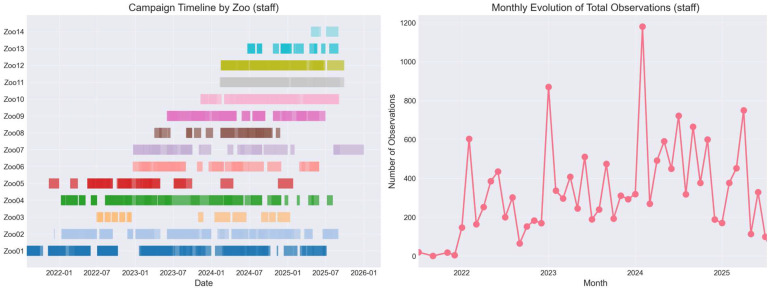
Observation effort over time across monitoring campaigns. Staff-only sessions are shown (excluding interns and external observers).

Observation sessions were distributed throughout the year using institution-specific “campaigns” aligned with operational schedules (Figure 9). This approach supported sustained behavioural monitoring, with an average observation effort ranging from approximately 0.3 to 1.1 sessions per day, depending on the institution. Monthly variation in observation intensity reflected differences in operational context and species coverage.

Taken together, these results illustrate both the capacity of the AWM protocol to generate welfare-relevant information at the individual and group levels, and the practical constraints associated with its long-term implementation in zoological institutions. These findings are discussed below in relation to existing welfare assessment frameworks, methodological limitations, and future perspectives.

## 4. Discussion

Building on the conceptual foundations of the Five Domains model and the operational principles of Welfare Quality^®^, Animal Welfare Monitor (AWM) was developed to address key limitations of existing welfare assessment tools in zoological settings, notably the scarcity of species-specific protocols and the operational complexity of generalised frameworks. Unlike approaches that require extensive prior biological expertise and may increase the risk of subjective interpretation [26], AWM provides tailored, structured questionnaires for each species, enabling consistent application across diverse taxa. By embedding species-specific knowledge directly within the assessments, AWM fulfils a pedagogical function, progressively supporting welfare literacy among animal care teams during routine use [25,28]. While several multi-species welfare assessment frameworks have recently been developed as alternatives to generic checklists, their application has typically been limited to a relatively small number of taxa. For example, the S-AWAG protocol has been tested on 11 species [31], the Zoo-Well approach on 12 species [35], and the Ackonc-AWA framework was applied to 14 species [25]. In contrast, with 87 species currently covered across mammals, birds, reptiles, and amphibians, AWM constitutes a substantial database of species-specific welfare assessment protocols embedded within a single, standardised methodological framework. This design supports zoological institutions in monitoring welfare across a broad taxonomic range while maintaining species-level biological relevance.

Validating welfare indicators in zoo contexts remains challenging due to limited sample sizes and uneven availability of behavioural and husbandry literature across taxa [27,39]. To address these constraints, the AWM protocol relies on a hierarchical framework with four levels of question specificity (base, order, family, species), allowing both methodological consistency and biological relevance [33]. This structure enables informed extrapolation from related taxa when species-specific data are scarce, while remaining adaptable as new evidence emerges. Continuous literature review, combined with iterative feedback from participating institutions, supports the progressive refinement of indicators and strengthens both the scientific robustness and practical relevance of the protocol [16].

The combined use of structured questionnaires and behavioural observations provides a multidimensional assessment of animal welfare. Questionnaire-based indicators focus primarily on environmental and management inputs known to influence affective states, while behavioural observations capture animal-based outcomes such as affiliative interactions, feeding behaviour, exploration, play, and abnormal behaviours [48]. Observer-related bias is mitigated through standardised training, calibration phases, and the explicit recording of behaviours directed towards observers [33]. Longitudinal monitoring further enables evaluation of welfare stability and changes over time, particularly in response to management interventions or social transitions. Both case studies should be viewed as illustrative demonstrations of the AWM framework applied under operational zoo conditions. They are descriptive in nature and are not intended to provide causal evidence or formal validation of welfare interventions, but rather to highlight feasibility, interpretability, and practical integration within existing management practices.

Beyond data collection, a central contribution of AWM lies in its capacity to translate assessment outputs into welfare-oriented decision-making. Rather than relying on isolated interpretations, the protocol promotes collective analysis of results by multidisciplinary teams, including keepers, veterinarians, biologists, and managers. This shared interpretation process reduces individual bias, integrates complementary expertise, and supports prioritising welfare actions based on urgency, feasibility, and expected impact. The iterative assessment–action–reassessment cycle implemented within AWM aligns with adaptive management principles and ensures that welfare monitoring remains a dynamic, outcome-oriented process rather than a static evaluation exercise.

Visual media constitute an important complementary component of this decision-support framework. Although photographs and videos do not directly influence welfare scores, they provide contextual information that enhances the interpretation of scores, facilitates communication among team members, and supports institutional memory. Visual documentation is particularly valuable for capturing seasonal variation, enclosure configurations, or gradual environmental changes that may not be fully reflected in numerical indicators alone. Consistent with previous findings, such visual records can reduce inter-observer variability and improve the transparency of welfare assessments [31,60]. More broadly, they help address a recurring challenge in applied welfare science: transforming complex datasets into actionable insights that can be readily shared with decision-makers [61,62,63].

Successful implementation of welfare assessment tools depends not only on methodological robustness but also on staff engagement and institutional integration [64]. The participatory design of the AWM framework, which actively involves animal care teams in data collection and interpretation, reflects the One Welfare framework by recognising the interdependence between animal welfare, human well-being, and organisational context [65]. Inclusive engagement fosters ownership of the assessment process, enhances data reliability, and supports the integration of welfare monitoring into daily routines. The autonomous use of the framework, combined with its availability in multiple languages, further facilitates adoption across institutions of varying sizes and resources. While the protocol has been implemented exclusively within EAZA-accredited zoos to date, its design and multilingual availability allow for straightforward adaptation and use by other regional zoo associations as a logical next step in its global development.

Despite these strengths, several limitations remain. Current AWM development shows a taxonomic bias toward mammals, particularly primates and carnivores, reflecting both the availability of research and institutional priorities. Expanding coverage to underrepresented taxa such as birds, reptiles, amphibians, and invertebrates remains a priority. While the four-level hierarchical structure has proven effective, broader application has revealed situations in which genus-level information may be more informative than family-level data yet insufficiently detailed at the species level. Incorporating genus as an intermediate tier could therefore refine indicator specificity without substantially increasing development workload.

From an operational perspective, sustaining the recommended assessment frequency proved challenging across institutions. Although behavioural observations were consistently maintained over multiple years, repeated completion of comprehensive, species-specific questionnaires required substantial time. Observation sessions were brief (10 min), whereas questionnaires required approximately 45 min plus additional time for multi-individual groups. These constraints are not unique to the AWM protocol and mirror limitations reported for other welfare assessment tools (Ackonc-AWA: 51.32 ± 29.36 min initial, 58.32 ± 23.11 min follow-up; plus potential activity budgets [25]) or shorter (C-Well^®^: two days per ten dolphins [34]). For other, more recently published species-specific protocols (e.g., [16,36]), implementation times remain unreported. Nonetheless, the challenge of sustaining repeated assessments over time is a known limitation of long-standing tools such as Welfare Quality^®^ [66] and C-Well^®^ [35].

In practice, reduced assessment frequencies—such as one or two evaluations per year or targeted pre- and post-event assessments—appear more feasible. For large social groups, individual-level assessments may be impractical, and group-level questionnaires, modelled on existing frameworks, could offer a pragmatic alternative. Indeed, group-level analysis might be applied to large groups that can exist in zoos and aquariums. Consistent with the Welfare Quality^®^ approach, group-level assessment would not rely on simple aggregation of individual scores, but rather on identifying the proportion of individuals affected within a group for each indicator [13,23]. Such an approach allows the detection of welfare issues that may concern a subset of animals without being masked by high average scores. Applied to zoological settings, group-level outputs could provide a complementary perspective to individual monitoring while remaining aligned with the non-compensatory logic of the AWM framework.

Behavioural monitoring emerged as a particularly robust and scalable component of the protocol. All participating institutions sustained observation efforts over several years, with modest daily workloads and broad staff participation. Seasonal fluctuations in observation intensity were observed, reflecting staffing patterns and operational demands. Anticipatory scheduling or temporary external support may mitigate these effects. In contexts where individual identification is difficult, alternative sampling strategies, such as scan sampling, may enhance applicability without compromising data quality.

Finally, the digital architecture of AWM addresses long-standing challenges in welfare data management, including data traceability, calculation errors, and long-term accessibility. Previous tools, such as Welfare Quality^®^, have been constrained by time constraints, data formatting issues, and human error, leading to increased use of digital tools and mobile applications to streamline welfare monitoring [67]. Several protocols have followed this direction, such as AWAG [30,68], which uses cloud-based software, and Dolphin-WET [14], which integrates mobile functionality for field use. In zoo environments, apps like ZooMonitor have also demonstrated their usefulness in enhancing behavioural observations and data management [59,63]. While a paper-based version remains available, it is more time-consuming.

In line with this trend, AWM was conceived from the outset as a digital tool. Its mobile format offers a user-friendly interface for entering both questionnaire data and behavioural observations, with features such as real-time synchronisation (with an offline mode available during data collection), automated calculations (e.g., time budgets), and integrated photo documentation to streamline the process and enhance usability. The AWM framework addresses a current gap by offering a species-specific, scalable tool that reduces the need for extensive retraining or new tool development for each species. Its digital structure improves data quality, traceability, and adaptability, enabling zoo teams to monitor welfare parameters more accurately—across devices and even in offline conditions—while fostering efficient, evidence-based management. This integration of digital tools, species-specific knowledge, and longitudinal data collection represents a significant step forward for applied welfare science, strengthening the connection between scientific research, welfare assessment, and day-to-day management practices.

## 5. Conclusions

The Animal Welfare Monitor tool represents a pragmatic and scientifically grounded advance in zoo animal welfare assessment. By operationalising the Welfare Quality^®^ principles across a broad taxonomic range—currently encompassing 87 species—the AWM framework bridges the gap between high-level ethical standards and daily husbandry practices. This standardised species-specific approach is particularly relevant for international population management programmes such as European Endangered Species Programmes (EEP) and Species Survival Plans (SSP), which rely on consistent welfare standards across institutions to support sustainable breeding, transfers, and long-term demographic stability. Good welfare is not only an ethical imperative but also a biological prerequisite for reproductive success, genetic diversity, and the viability of managed populations intended to contribute to conservation goals.

Beyond population management, AWM also provides a structured framework to support institutional research and education. The standardisation of welfare assessments facilitates data comparability over time and across facilities, thereby enabling retrospective analyses, collaborative research initiatives, and the integration of welfare indicators into applied conservation science. In addition, transparent and evidence-based welfare monitoring strengthens the educational mission of zoos by allowing institutions to demonstrate their commitment to animal care, scientific rigour, and accountability to the public and regulatory bodies.

The inclusion of visual records and a mobile-friendly format improves traceability, team communication, and long-term follow-up. This solution helps animal care professionals to track, assess, and enhance welfare over time with clarity and efficiency. By integrating digital tools, species-specific knowledge, and longitudinal monitoring into a single framework, AWM contributes to the advancement of applied welfare science by strengthening the link between scientific research, welfare assessment, and day-to-day management practices in zoological settings.

While further refinements remain necessary—particularly regarding group-level assessment strategies and the expansion of taxonomic coverage—AWM provides a scalable and flexible tool to support welfare improvements across a wide range of species and institutional contexts. Its ongoing development, informed by scientific advances and feedback from zoo professionals, will be essential to maintaining its relevance and long-term impact within zoological institutions. Ultimately, AWM aims to support the global zoological community in advancing conservation, research, and education while ensuring the highest possible quality of life for animals under human care.

## Figures and Tables

**Table 1:** Criteria selected for the AWM protocol for the zoological context, across the four welfare principles, adapted from Welfare Quality^®^. Each principle is broken down into measurable criteria that allow for a multidimensional evaluation of the animal’s welfare state.

Principles	Welfare Quality^®^ Criterion	Welfare Quality^®^ Measure	AWM Criterion	AWM Measure
HOUSING	- Comfort around resting	Time needed to lie down, cleanliness of animals	- Indoor enclosure design - Cleanliness of the environment - Visual comfort	Environmental cleanliness, behavioural observations, enclosure layout, substrates, light transition
	- Thermal comfort	No current measure	- Thermal comfort	Choice of climate zones
	- Ease of movement	Pen features according to live weight, access to an outdoor loafing area in pasture	- Surface - Outdoor enclosure design - Behavioural possibilities—indoor - Behavioural possibilities—outdoor - Safety and prevention	Space available, enclosure layout, risks for the animals, possibility of hiding
NUTRITION	- Absence of prolonged hunger	Body Condition Score	- Diet composition	Food intake, frequency of weight measurements,
	- Absence of prolonged thirst	Water provision, cleanliness of water points, number of animals using water points	- Food presentation - Hygiene and preparation	Frequency and methods of feeding, cleanliness of the feeding areas and water points, water provision
HEALTH	- Absence of injuries	Lameness, integument alterations	- Physical condition	Body condition, wound, lameness, coat, skin or feathers condition, coughing, nasal discharge, ocular discharge, hampered respiration, diarrhoea
	- Absence of disease	Coughing, nasal discharge, ocular discharge, hampered respiration, diarrhoea, bloated rumen, mortality	- Preventive monitoring	Prophylaxis programme, weight monitoring
	- Absence of pain induced by management procedures	Disbudding/dehorning, tail docking, castration	- Curative care	Curative treatments
BEHAVIOUR	- Expression of social behaviours	Agonistic behaviours, cohesive behaviours	- Expressed behaviours	Social group composition, cohesive behaviours, agonistic behaviours,
	- Expression of other behaviours	Access to pasture	- Enrichments and stimulations	Interests and use of enrichments and stimulations,
	- Good human–animal relationships	Avoidance distance	- Training	Willingness to participate in training, response to caretakers and to visitors
	- Positive emotional state	Qualitative behaviour assessment	- Choice and control - Behavioural monitoring	Choice and control, free access, diversity of behaviours expressed

**Table 2:** Description of the five-level scoring system for management-based, resource-based, and animal-based indicators. The scale is designed to identify both urgent welfare risks and opportunities for positive welfare enhancement.

	Score −1	Score 0	Score 1	Score 2	Score 3
	Negative	Concerning	Acceptable	Positive	Fulfilled needs
**Management-based**	Practice has a clearly negative impact on welfare and should be modified or eliminated urgently.	Practice may not meet current best practices and should be reviewed or improved.	Practice is functional and does not compromise welfare, though improvements may be possible.	The practice supports welfare proactively and aligns well with established recommendations.	The practice is fully dedicated to ensuring species’ needs, demonstrating innovation or excellence in welfare promotion.
**Resource-based**	The resource is inadequate, absent, or unsuitable, representing a significant risk to welfare.	The resource is present but suboptimal, offering limited benefit or functionality.	The resource meets minimum requirements and supports baseline welfare.	The resource is appropriate, accessible, and enhances the animal’s ability to express natural behaviours.	The resource is of excellent quality, standards, highly enriching, and tailored to the species’ specific needs.
**Animal-based**	Clear evidence of compromised welfare (e.g., injury, stereotypy, abnormal posture).	Signs suggest a potential welfare issue; monitoring or further investigation is needed.	No sign of distress or suffering; the animal’s state is within acceptable welfare parameters.	Evidence of good welfare, such as normal behaviour patterns or signs of comfort.	Clear expression of positive welfare states (e.g., affiliative interactions, engaging with the environment).

**Table 3:** Examples of similarities and differences within the behavioural category “Exploration and locomotion” in the ethograms for (**A**) giraffes and (**B**) Northern ground hornbills. Behaviours marked with an asterisk (*) are shared between the two ethograms.

(A) Giraffe: Exploration and locomotion behaviours	
Behaviour	Definition
Walking	Moving forward or backwards on its four legs at a slow/moderate pace. Excluding rumination.
Galloping	Moving on its four legs at a rapid pace.
Exploring *	Moving at an irregular pace, sniffing or touching/brushing elements in its environment with its mouth, legs, tongue, head. May include flehmen (not directed at a conspecific): the head is raised and the upper lip is curled. Excluding handling of objects or food.
Manipulating a non-food object or playing alone *	Manipulates an object by pushing or hitting it with its forehead, muzzle, tongue, or legs. Gallops, leaping, shaking the head, arching the back.
Pacing *	Walking the same path repeatedly, which may involve a specific path (such as a circle, back and forth, or repetitive movement sequence).
**(** **B) Northern ground hornbill: Exploration and locomotion behaviours**	
**Behaviour**	**Definition**
Walking on the ground	Moving on both feet at a slow/moderate pace on the ground.
Moving above ground level	Moving on a raised structure (perch, platform, etc.).
Running	Moving on both feet at a rapid pace on the ground. The wings may remain folded or open slightly to maintain balance, but they do not flap to generate flight.
Jumping	On both feet, fixing its gaze on the landing site, leaning forwards to gain speed and jumping off, flapping its wings.
Flying	Moving through the air by flapping its wings or hovering.
Rummaging in the substrate or vegetation	Using its claws or beak to move vegetation or scratch in the substrate.
Exploring *	Moving at an irregular pace, visually inspecting the environment (e.g., tilting head). Excluding the manipulation of objects or food and moving substrate and vegetation.
Manipulating a non-food object or playing alone *	Rolling an object, pulling on an object, taking it in its beak, etc. Excluding moving with an object in its beak.
Carrying a non-food object	Moving around with an object in its beak.
Building, setting a nest	Being near or in the nest. Incorporating branches or substrate into the nest or repositioning a nest element.
Pacing *	Walking the same path repeatedly, which may involve a specific path (such as a circle, back and forth, or repetitive movement sequence).

**Table 4:** Analytical behavioural categories used for time-budget analysis and welfare interpretation.

Main Category	Analytic Category	Behaviour Examples
Exploration and locomotion	Exploration and locomotion	Walking, jumping, manipulating a non-food object
	Abnormal behaviours	Pacing
Social behaviours	Social—positive	Positive without contact, playing with conspecifics
	Social—negative	Negative with or without contact
	Social—undetermined	Other interaction undetermined
Feeding behaviours	Feeding	Manipulating food or an object containing food, drinking
	Abnormal behaviours	Eating abnormal items
Individual behaviours	Observation	Observing (towards environment or conspecifics)
	Vigilance	Vigilant (towards environment or conspecifics)
	Rest	Resting
	Grooming and maintenance	Grooming, scratching
	Abnormal behaviours	Repeated head movements (giraffe), licking surface (giraffe), repeatedly opening wings without flying off (NGH)
Directed towards the observers	Observation	Observing the observer
	Vigilance	Vigilant towards the observer
	Human–animal interaction	Seeking the observer’s attention
Other	Human–animal interaction	Seeking attention from humans (visitors, staff members, etc.).
	Other species interaction	Positive or negative interactions towards other species
	Undetermined	Unknown/off-list behaviour
	Not visible	Not visible

**Table 5:** Taxonomic representativeness of AWM database, showing the diversity of the 87 species-specific protocols currently operationalized across different animal classes. The number of species per taxa is indicated between parentheses.

Family	Mammals (69 Species)	Birds (15 Species)	Reptiles (1 Species)	Amphibians (2 Species)
**Order**	Diprotodontia (1)	Casuariiformes (1)	Squamata (1)	Anura (2)
	Proboscidea (1)	Sphenisciformes (1)		
	Pilosa (1)	Accipitriformes (1)		
	Primates (37)	Strigiformes (2)		
	Rodentia (1)	Bucerotiformes (2)		
	Carnivora (13)	Falconiformes (1)		
	Perissodactyla (5)	Psittaciformes (5)		
	Cetartiodactyla (10)	Cariamiformes (1)		
		Gruiformes (1)		

**Table 6:** Number of species monitored per zoological institution using the AWM protocol.

Institution	Number of Species	Institution	Number of Species
**Zoo 01**	66	Zoo 10	12
**Zoo 04**	21	Zoo 11	11
**Zoo 07**	21	Zoo 06	10
**Zoo 02**	20	Zoo 13	10
**Zoo 12**	16	Zoo 08	7
**Zoo 03**	15	Zoo 14	6
**Zoo 09**	15	Zoo 05	4

**Table 7:** Frequency of questionnaire assessments per species per year across institutions.

Species Annual Evaluation Frequency	1×/Year	2×/Year	3×/Year	4×/Year
**2022**	31%	22%	35%	12%
**2023**	58%	30%	10%	1%
**2024**	63%	15%	22%	0%

## Data Availability

The raw data supporting the conclusions of this article will be made available by the authors on request.

## Supplementary Materials

The following supporting information can be downloaded at: https://www.mdpi.com/article/10.3390/ani16050842/s1, Table S1: Repartition of type of indicators (animal-based, resource-based, management-based) in each principle; Table S2: Examples of questionnaire items assigned a weighting coefficient of 2; Table S3: Sensitivity analysis; Table S4: Complete ethograms for giraffes and Northern ground hornbills; Table S5: Example of inter-observer reliability; Table S6: Full list of species covered by AWM; Figure S1: Seasonal enclosure variation (greater kudu); Figure S2: Quarterly assessment frequency per institution; Figure S3: Comparison of the number of observations between staff only and all participants. Supplementary Methods S1: Calculation of indicator, criterion, and principle scores; Supplementary Methods S2: Inter-observer reliability and calibration procedures.

## Abbreviations

The following abbreviations are used in this manuscript:

EAZA	European Association of Zoos and Aquaria
AZA	Association of Zoos and Aquariums
PAAZA	Pan-African Association of Zoos & Aquaria
