# Supplementary material for: Animal Welfare Monitor: Raising the Bar for Species-Specific Welfare Evaluation Using Welfare Quality® Principles
Source: Animals (Basel). 2026 Mar 7;16(5):842. doi: 10.3390/ani16050842 (PMC12984143; doi:10.3390/ani16050842)
Supplement: Supplementary file 1 [file animals-16-00842-s001.zip › Supplementary Materials.pdf]

Supplementary materials

Table S1. Repartition of the 81 welfare indicators defined within the AWM framework, including 21 animal-based, 31 resource-based, and 29 management-based indicators.

Principles	Animal-based (outputs)	Resource-based (inputs)	Management-based (inputs)
Housing	0	23	6
Nutrition	0	4	9
Health	10	0	4
Behaviour	11	4	10
**Total**	**21**	**31**	**29**

Table S2. Practical examples of high-impact welfare indicators (coefficient 2) and their biological justification within the scoring system. This table clarifies how certain critical health and behavioral factors are prioritized to ensure they have a decisive impact on the categories score.

Welfare Principle	Question	Justification for Coefficient 2
Feeding	"How are species-specific characteristics taken into account in food presentation?"	Prioritises the expression of highly motivated species-specific behaviours (e.g., specific postures or substrates). The fulfilment of these behaviours is crucial for psychological well-being.
Health	"If the animal shows symptoms, is the veterinarian informed?"	Ensures a rapid and adequate clinical response or diagnosis when symptoms are detected. Timely professional assessment is vital to mitigate acute health risks.
Health	"Does the overall physical condition (e.g., weight, injury, disability) impact the animal's behaviour?"	Recognises that physical limitations restricting natural behavioural expression have a high potential impact on the individual's global welfare state.
Housing	"Can the animal hide within the enclosure?"	Promotes individual choice and control. The ability to withdraw from the sight of congeners or visitors is essential to manage potential social or environmental stressors.

Table S3. Sensitivity analysis of AWM category scores across alternative weighting scenarios**.** Sensitivity effects were quantified as the difference (Δ) between scenario scores and baseline scores, expressed in percentage points. Category-level welfare scores are shown for the baseline weighting scheme and two alternative scenarios (equal weighting, increased weighting) to evaluate the stability of welfare interpretations under different weighting assumptions.

Individual	Category	Baseline (%)	Δ Equal Weights	Δ High Impact 3.0
Ind.1	Nutrition	88.88	−0.59	+0.53
	Behaviour	55.94	−2.64	+2.28
	Housing	58.25	+6.74	−5.35
	Health	75.69	+2.26	−1.49
Ind.2	Nutrition	92.39	−0.42	+0.37
	Behaviour	62.68	−1.48	+1.31
	Housing	55.52	+6.90	−5.60

	Health	71.54	+1.26	−0.82
Ind.3	Nutrition	92.38	−0.44	+0.39
	Behaviour	57.75	−1.82	+1.59
	Housing	56.42	+7.29	−5.85
	Health	77.42	+1.40	−0.90

Because weighting procedures can influence score magnitude and interpretation, a sensitivity analysis was conducted to evaluate the robustness of the AWM framework to alternative weighting assumptions. The sensitivity analysis was conducted using empirical data collected for three giraffes housed in a zoological institution, with a total of 34 completed questionnaires were available, spanning multiple assessment dates.

Two alternative weighting scenarios were evaluated and compared to the baseline:

1. Equal Weights: all questions assigned a weight of 1.0.
2. High Impact 3.0: key indicators assigned a weight of 3.0 instead of 2.0.

For each scenario, category scores were recalculated using the same normalisation procedure (0–100 scale). Sensitivity effects were quantified as the difference (Δ) between scenario scores and baseline scores, expressed in percentage points.

Finally, the digital architecture of the AWM addresses long-standing challenges in welfare data management, including data traceability, calculation errors, and long-term accessibility.

Table S4. Complete ethograms for giraffes and Northern ground hornbills.

Table S5. Example of inter-observer reliability. For each dyad, the percentage of agreement during the session, the kappa coefficient and the ICC for the session has been calculated. Inter-observer agreement was quantified using three complementary metrics: Percentage of agreement, representing the percentage of second-by-second observations in which both observers recorded the same behavioural state; Kappa coefficient, corresponding to Cohen’s kappa coefficient accounting for chance agreement; and ICC (session), representing the intraclass correlation coefficient calculated for the aggregated 10-minute observation sequence.

Animal	Test number	Observer ID_A	Observer ID_B	Percentage of agreement	Kappa coefficient	ICC (session)
Ind1	Test 1	Observer1	Observer2	96,6	0,883	0,989
Ind2	Test 2	Observer1	Observer2	95,5	0,924	0,966
Ind3	Test 3	Observer1	Observer2	74,4	0,0298	0,0566
Ind3	Test 1	Observer3	Observer4	61,3	0,353	0,432
Ind3	Test 2	Observer3	Observer4	71,2	0,609	0,69
Ind4	Test 1	Observer5	Observer1	44	0,344	0,316
Ind3	Test 3	Observer3	Observer4	80,2	0,721	0,595
Ind5	Test 4	Observer1	Observer2	90,3	0,465	0,542
Ind3	Test 4	Observer3	Observer4	99,7	0,943	0,992
Ind3	Test 5	Observer3	Observer4	80,5	0,666	0,492
Ind3	Test 1	Observer2	Observer4	79,9	0,593	0,755
Ind3	Test 1	Observer4	Observer5	92,1	0,404	0,724

Analyses were conducted in R using the following packages and purposes: *openxlsx* (import of Excel observation exports), *janitor* (standardizing and cleaning variable names), *tidyverse*—including *dplyr*

(data cleaning and wrangling), *tidyr* (reshaping data and expanding event records to second-by-second series), *purrr* (iterating computations across observer dyads/triads), and *ggplot2* (visualization and diagnostic plots)—as well as *lubridate* (handling Unix timestamps and date–time operations). Inter-rater reliability was assessed with *irr* (Cohen's κ and ICC calculations) and *psych* (additional reliability outputs and cross-checks when needed).

Table S6: Full list of species covered by AWM.

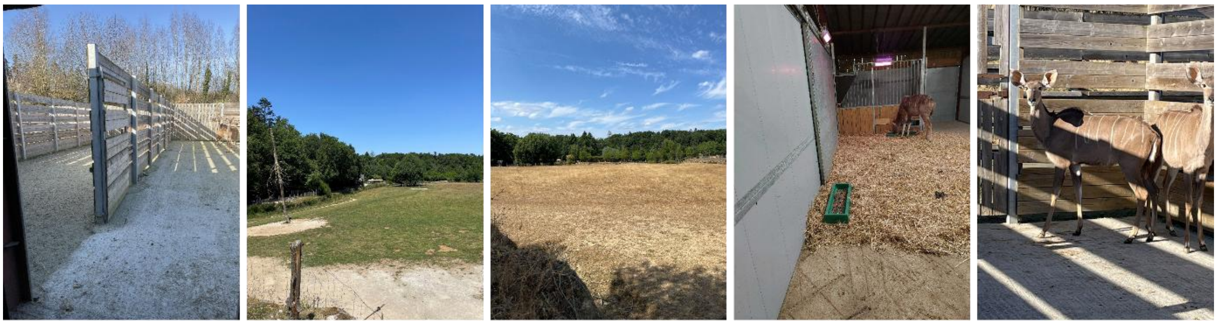

January 2022 June 2022 August 2022 November 2022 February 2023

Figure S1. A series of five images taken over different seasons in 2022 and 2023, documenting environmental changes in the outdoor enclosure of a group of greater kudus.

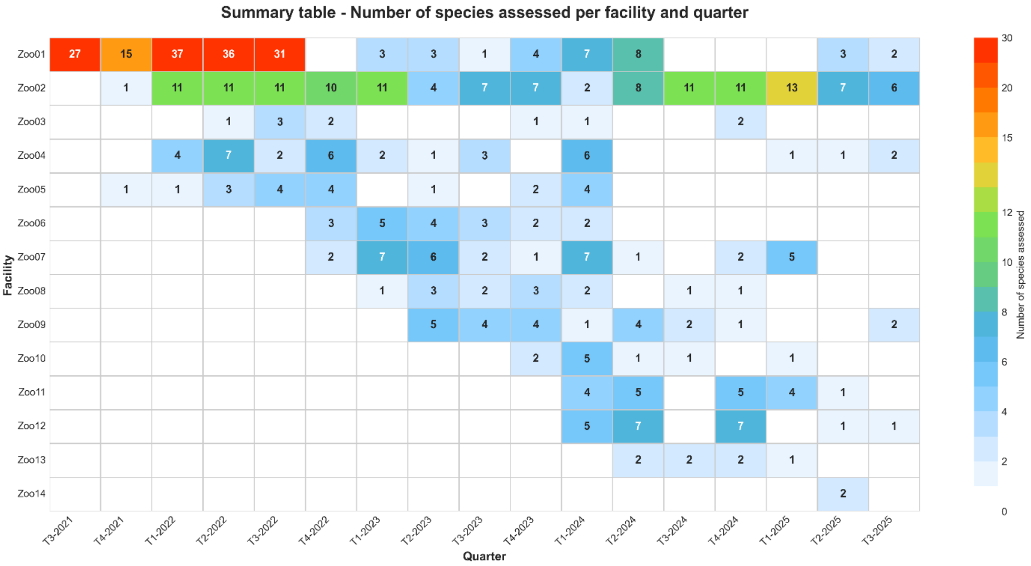

Figure S2. Number of species assessed per institution and per quarter, from 2021 to 2025.

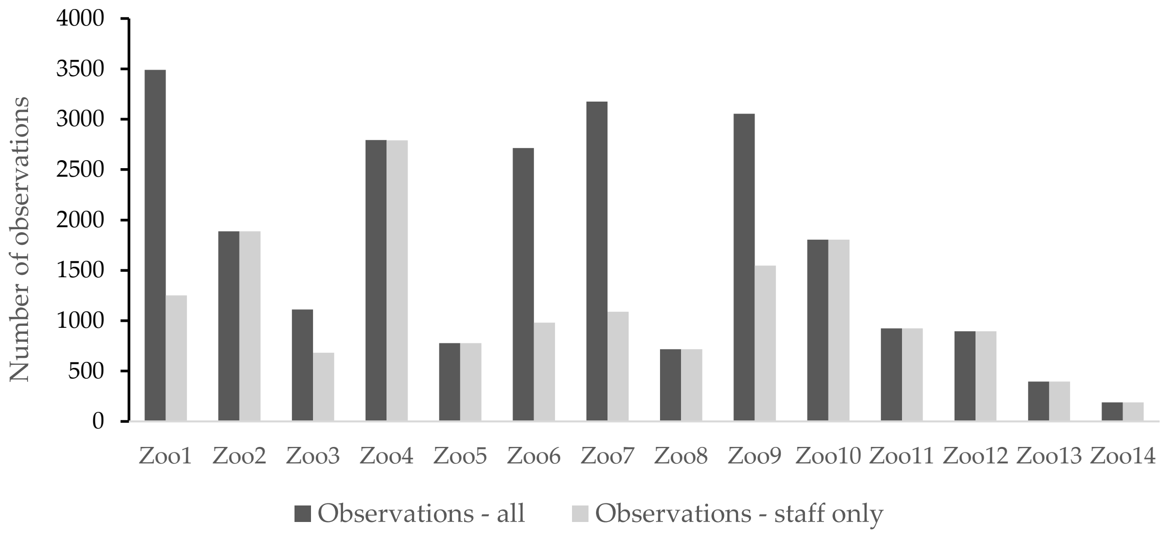

Figure S3. Comparison of the number of observations between staff only and all participants, for each zoo. Dark grey bars: all observations made by zoo staff, including external researchers and interns. Light grey bars: permanent zoo staff only.

Supplementary Methods S1 - Calculation of indicator, criterion, and principle scores

Each questionnaire item is treated as an indicator and is scored on the five-level scale described in Table 2, yielding an indicator score S_i (range: -1 to $+3$). Each indicator i is assigned a weighting coefficient W_i (default = 1; higher values for indicators considered more welfare-relevant). Weighted indicator scores are aggregated to compute criterion and principle scores.

For each indicator i, the adjusted (weighted) score is:

$$AS_i = W_i \times S_i$$

where:

- AS_i = adjusted (weighted) score for indicator i
- S_i = raw indicator score (Table 2)
- W_i = weighting coefficient for indicator i

A criterion c comprises n_c indicators. Let $I_c \subseteq \{1, \dots, n_c\}$ denote the set of indicators included in the computation for criterion c, i.e., indicators for which all selected response option scores are defined (no non-computable/non-applicable values among selected options). Only indicators in I_c contribute to criterion scoring.

The total weighted points obtained for criterion c are:

$$P_c = \sum_{i \in I_c} A\,S_i = \sum_{i \in I_c} W_i\,S_i$$

criterion c comprises n_c indicators. Let $\mathcal{I}_c \subseteq \{1, \ldots, n_c\}$ denote the set of indicators for criterion c

The total weighted points obtained for criterion c are:

$$P_c = \sum_{i \in \mathcal{I}_c} A\, S_i = \sum_{i \in \mathcal{I}_c} W_i\, S_i$$

For each indicator i, let $S_i^{\max}$ denote the maximum achievable score given the indicator's response options (and item-level caps, when defined; see below). The total weighted maximum for criterion c is:

$$M_c = \sum_{i \in \mathcal{I}_c} W_i\, S_i^{\max}$$

The criterion score (percentage) is then computed as:

$$S_c(\%) = \left\lceil 100 \times \frac{P_c}{M_c} \right\rceil$$

and truncated at zero if negative:

$$S_c(\%) = \max\left(S_c(\%), 0\right)$$

If $M_c = 0$, the score is undefined (returned as null in the software).

Item-level caps for multiple-choice indicators. For multiple-choice indicators where several options can be appropriate but not simultaneously applicable, the maximum achievable score may be capped at the item level. In such cases, $S_i^{\max}$ is set to the predefined cap, and the obtained indicator score is also capped accordingly:

$$S_i \leftarrow \min\left(S_i, U_i\right), S_i^{\max} \leftarrow U_i$$

where U_i is the item-level maximum (when specified). If an item-level minimum L_i is defined, then:

$$S_i \leftarrow \max\left(S_i, L_i\right)$$

A welfare principle p (i.e. Housing, Nutrition, Health, Behaviour) comprises m_p criteria. Principle scores are computed by aggregating criterion scores.

If all criteria are equally weighted within a principle:

$$PS_p(\%) = \frac{1}{m_p}\sum_{c=1}^{m_p} S_c\,(\%)$$

If criteria are assigned criterion-level weights V_c (optional; default $V_c = 1$):

$$PS_p(\%) = \frac{\sum_{c=1}^{m_p}(V_c \times S_c(\%))}{\sum_{c=1}^{m_p} V_c}$$

To prevent compensation between domains, principle scores are reported separately and are not combined into a single overall welfare score. This ensures that a low score in one principle cannot be masked by high scores in another.

Supplementary Methods S2: Inter-observer reliability and calibration procedures

1. Recording alignment. Observers conducted paired live focal sessions on the same individuals using the same ethogram. Behavioural labels were recorded continuously and aligned at 1-s resolution over 10-min sequences (600 time points).
2. Second-by-second agreement. We computed (i) percent agreement (proportion of seconds assigned to the same behavioural category) and (ii) Cohen's κ from the second-by-second contingency table to quantify chance-corrected agreement. κ values were interpreted using commonly used thresholds: <0.20 slight, 0.21–0.40 fair, 0.41–0.60 moderate, 0.61–0.80 substantial, >0.80 almost perfect.
3. Agreement on time allocation. To test agreement in time spent per behaviour, second-by-second labels were aggregated into 30-s windows. For each behaviour and window, we computed the number of seconds allocated by each observer and quantified agreement using a two-way, absolute-agreement ICC (single measure). ICC was interpreted as poor (<0.50), moderate (0.50–0.75), good (0.75–0.90), excellent (>0.90).
4. Validation decision rules. Dyads were considered validated when κ ≥ 0.61 and ICC ≥ 0.75. Dyads with ICC 0.60–0.75 were considered conditionally acceptable only when κ met the validation threshold and discrepancies were attributable to brief transition timing differences rather than systematic behavioural misclassification; targeted discussion and re-testing were then implemented.
5. Live observation constraint. Because observations were conducted live (field use), sequences could not be replayed for post-hoc adjudication. Reliability was therefore quantified under operational conditions using paired sessions and iterative calibration when thresholds were not met.
6. Networked calibration approach. When it was not feasible to test all observer pairings, each observer was required to meet the above thresholds with at least one trained anchor observer who had demonstrated agreement with multiple team members, supporting consistency across larger teams despite incomplete pairwise testing.
