# Supplementary material for: Animal Welfare Monitor: Raising the Bar for Species-Specific Welfare Evaluation Using Welfare Quality® Principles
Source: Animals (Basel). 2026 Mar 7;16(5):842. doi: 10.3390/ani16050842 (PMC12984143; doi:10.3390/ani16050842)
Supplement: Supplementary file 1 [file animals-16-00842-s001.zip › Table S4_Ethogram_Giraffe.pdf]

Ethogram - Giraffe

Behavioural category	Behaviour	Definition
Exploration and locomotion	Walking	Moves forward or backwards on its four legs at a slow/moderate pace
	Galloping	Moves on its four legs at a rapid pace
	Exploring	Moves at an irregular pace, sniffing or touching/brushing elements in its environment with its mouth, legs, tongue, head. May include flehmen (not directed at a conspecific): the head is raised and the upper lip is curled. Excludes handling of objects or food.
	Manipulating a non-food object or playing alone	Manipulates an object, pushes or hits it with its forehead, muzzle, tongue or legs. Gallops, leaping, shaking the head, arching the back. Can be expressed near a conspecific but not directly towards conspecifics (e.g. no contact)
	Pacing	Walking the same path repeatedly, which may include a specific path (in a circle, back and forth, repetitive movement sequence, etc.)
Social behaviours	Playing with conspecifics	Chasing after a positive interaction between two individuals, simulating a fight, with reciprocal interactions between the two conspecifics, etc.
	Mating	The male mounts the female from behind, his front legs on each side of her flanks. May or may not result in intromission
	Positive WITHOUT contact	Follows a conspecific, vocalizes, sniffs a conspecific without contact. May include flehmen in proximity: the head is raised and the upper lip is curled
	Positive WITH contact	Mutual grooming, rubbing against a conspecific, etc.
	Social rest	Rest (eyes half-closed or closed, relaxed attitude, standing or lying down) in contact with or less than two meter away from one or more conspecifics. Excludes rumination
	Negative WITHOUT contact	Headbutt or foot thrown in the direction of a conspecific, charges, chases, etc.
	Negative WITH contact	Headbutt, kick, etc.
	Object or food stealing	Taking or having its place taken over at a food source or an object
	Avoiding a conspecific or submitting	Moving aside or away when a conspecific approaches, changing trajectory, hiding, etc.
	Other interaction, neutral or undetermined	Social interaction with a conspecific that is not on the list or whose function has not been identified.
Feeding behaviours	Drinking	Spreading the front legs, head down toward the water trough or watering hole, and swallowing the water (or other drink)
	Ruminating	Regurgitating then chewing with regular movements and swallowing food while lying or standing, relaxed attitude
	Manipulating food or an object containing food	Manipulating food or an object containing food with its tongue or foot, eating food contained inside the object
	Eating hay	Taking fodder (hay, alfalfa, etc.) with its teeth or tongue, chewing and swallowing it
	Eating branches	Tearing up branches or leaves (trees or shrubs foliage, branches, etc.) with its teeth or tongue, chewing and swallowing them.
	Eating other foods (pellets, vegetables, etc.)	Ingesting fodder or other foods (pellets, vegetables, etc.)
	Licking a salt stone	Licking or ingesting a food supplement in the form of a block or salt stone

	Grazing	Tearing up low vegetation (grasses, ferns, young shoots, etc.) with its teeth or tongue, chewing it and swallowing it
	Eating food given by visitors	Ingesting food given or thrown by visitors
	Chewing	Mastication not related to feeding or rumination
	Salivating	Liquid flows from the animal's mouth, possibly after regurgitation of stomach contents. Can be still or moving
	Eating abnormal items (substrate, excrement, etc.)	Ingesting elements that are not part of its diet: substrate, excrements, etc.
**Individual behaviours**	Observing (environment, conspecifics)	Looking at the environment or at conspecifics (no object manipulation or exploration). Relaxed posture
	Vigilant (environment, conspecifics)	Looking at the environment or at conspecifics (no object manipulation or exploration). Relaxed posture
	Inactive, resting	Standing or lying down, in a relaxed attitude, eyes half-closed or closed. Lying down, the head can be placed on its back. Excluding rumination
	Grooming, stretching, scratching	Licking a part of its body with its tongue. Extendings limbs (rear or front) and back. Scratching with a leg or the head
	Rubbing on a structure	Scratching a part of its body against an enclosure element (e.g. brush, rack, trunk, etc.)
	Thermoregulatory behaviour	Only for shivering movements (tremors, involuntary muscle contractions)
	Startled	Sudden movement of the body (e.g. following a sudden noise)
	Urinating, defecating	Urine or feces emission
	Licking a surface	Repeatedly rubbing its tongue and/or mouth on a surface (structure, wall, etc.). Repeatedly using its tongue to lick non-food surfaces/materials (metal bars, trunk, etc.)
	Repetitive head movements or other abnormal behavior	Repetitive head movements (vertical, horizontal, in a circle); Wagging its tongue in the air
**Directed towards the observer**	Observing the observer	Looking at the observer, following them with its eyes. The posture is relaxed. Standing, lying down, or moving
	Vigilant towards the observer	Looking at the observer, following them with its eyes. The posture is tense. The ears are erected and pointing towards the observer. The animal seems ready to flee or charge. Standing or moving
	Threatening	Kicking towards the observer, charging, etc.
	Running away, hiding	The animal tries to move away and hide from the observer
	Seeking the observer's attention	The animal approaches and seeks the observer's attention or contact, stretches its snout towards the observer, etc.
**Other**	Aimed at humans	Seeking attention, avoiding or threatening humans in the environment (visitors, staff members, etc.)
	POSITIVE towards other species	Positive social behaviors with another species (mutual grooming, food sharing, etc.)
	NEGATIVE towards other species	Negative social behaviors with another species (charging, avoiding, etc.)
	Undetermined towards other species	Social interaction with another individual that is not on the list or whose function has not been identified
	Off-list behaviour	Behaviour that does not correspond to any of the behaviours on the list (e.g.: avoiding an area or an object, shaking oneself)
	Not visible	The animal is not easily observable or not visible at all
	Unknown vocalization	Vocalizations whose function is not known
	Unknown behaviour	Unidentified behaviour
