# Supplementary material for: Animal Welfare Monitor: Raising the Bar for Species-Specific Welfare Evaluation Using Welfare Quality® Principles
Source: Animals (Basel). 2026 Mar 7;16(5):842. doi: 10.3390/ani16050842 (PMC12984143; doi:10.3390/ani16050842)
Supplement: Supplementary file 1 [file animals-16-00842-s001.zip › Table S4_Ethogram_Northern ground hornbill.pdf]

Ethogram - Northern Ground Hornbill

Behavioural category	Behaviour	Definition
Exploration and locomotion	Walking on the ground	Moving on both feet at a slow/moderate pace on the ground
	Moving above ground level	Moving on a raised structure (perch, platform, etc.)
	Running	Moving on both feet at a rapid pace on the ground. Wings can be spread out
	Jumping	On both feet, fixing its gaze on the landing site, leaning forwards to gain speed and jumping off, flapping its wings
	Flying	Moving through the air by flapping its wings or hovering
	Perching	Settling on a high element. Transitional behaviour: once the animal is perched, note what it does above ground level (e.g. observing, grooming itself, etc.)
	Rummaging in the substrate or vegetation	Using its claws or beak to move vegetation or scratch in the substrate
	Exploring	Moving at an irregular pace, visually inspecting environment (e.g., tilting head), may involve scratching or interacting with structures present. Excluding manipulating objects or food, moving substrate and vegetation
	Manipulating a non-food object or playing alone	Rolling an object, pulling on an object, taking it in its beak, etc. Excludes moving with an object in its beak.
	Carrying a non-food object	Moving around with an object in its beak
	Building, setting a nest	Being near or in the nest. Incorporating branches or substrate into the nest or repositioning a nest element
	Pacing	Walking the same path repeatedly, which may include a specific path (in a circle, back and forth, repetitive movement sequence, etc.)
Social behaviours	Positive WITHOUT contact	Begging for food, sharing food or nesting material without contact, etc. Excluding play and social rest
	Positive WITH contact	Mutual grooming (manipulating the feathers of another hornbill with its beak or being groomed), beak-to-beak food sharing
	Social rest	In resting phase (eyes half-closed or closed, relaxed attitude or even head turned and beak under the wing) in contact with or less than 1m away from one or more individuals
	Playing with conspecifics	Slow fight between two individuals. The interaction is reciprocal. The two individuals grab each other's beak, head or neck. Individuals lie down or chase each other by hopping with wings spread out. The game may involve an object that the individuals manipulate with their beak, steal from each other or exchange, etc.
	Negative WITHOUT contact	Lifting the head up to expose neck skin, then may lower its head and open its beak if the other conspecific approaches, taping its beak against a solid object, sometimes accompanied by rapid opening of wings, vocalizing, chasing, aggression attempts
	Negative WITH contact	Grabbing a conspecific's beak and twisting its head, charging brutally with beaks or feet strikes, jumping on a conspecific
	Territorial vocalisation (singing)	Several individuals emit a series of low-pitched “ooh...uh-uh-uh” sounds. The throat is inflated and deflated with each new series
	Beak slapping	Rubbing / slapping each other's beak
	Offering an object or food	Carrying an object, food or substrate (leaves, sticks, mulch, etc.) in its beak and presenting it to the other individual
	Provoking avoidance or submission	At its approach, or after a negative interaction, causes a conspecific to leave. Includes stealing objects/food and stealing space in an enclosure area (e.g. shelter, feeding).
	Avoiding a conspecific or submitting	Moving away when approached by a conspecific or after a negative interaction (pecking, kicking, etc.)
	Sexual behaviours	Attempted mounting or mounting of a conspecific with or without intromission.
	Other interaction, neutral or undetermined	Social interaction with a conspecific that is not on the list or which function has not been identified
Feeding behaviours	Carrying food	Moving with food in the beak
	Manipulating food or an object containing food	Peeling, flipping, moving, inspecting for a long time (several seconds), etc. of food or an object containing food
	Eating (e.g. meat, carcasses, insects, etc.)	Taking the food in the beak, then swallowing it after a series of head movements to bring it down into the beak
	Drinking	Leaning over, beak in contact with water (or other drink) and lifting the head to swallow. Can stand in water, but does not bathe

Behavioural category	Behaviour	Definition
Feeding behaviours	Eating food given by visitors	Taking food given or thrown by visitors into the beak, then swallowing it after a series of head movements to bring it down into the beak
	Eating abnormal items (substrate, wood, excrement, etc.)	Taking into the beak and ingesting elements that are not part of its diet: substrate, wood, excrement, etc.
Individual behaviours	Observing (environment, conspecifics)	Looking at the environment or at conspecifics (no object manipulation or exploration). Relaxed posture
	Vigilant (environment, conspecifics)	Looking at the environment or at conspecifics (no object manipulation or exploration). Tense posture, the animal seems ready to flee or attack, standing or moving
	Resting	Standing or lying down, eyes half-closed or closed, relaxed attitude; no reaction to surrounding noise/activity, with or without the head up
	Sunbathing	In the sun, lying flat on the ground, wings outstretched, tail fanned, head tilted to one side
	Taking a dust bath	Scratching the ground to detach the substrate, then lies down. Flapping its wings vigorously against the ground and agitating itself, scattering sand or dust over its body
	Taking a bath	Shaking its feathers and tail in the water, while raising its beak and spreading the water over its back, then flapping its wings OR lying down in the wet vegetation
	Grooming, stretching, scratching	Running its beak and/or lightly pinching its feathers to smooth them. Rubbing its beak on each side of a structure. Puffing up feathers, shaking body briefly, then spreading wings. Rubbing its skin with the tip of its beak or foot
	Yawning	Opening the beak wide and closing it quickly
	Startled	Sudden movement of the body (e.g. following a sudden noise)
	Thermoregulatory behaviour	Refering only to the following behaviours: panting (open beak, rapid throat movements, accompanying opening of wings, liquid may flow from nostrils); shivering (trembling, involuntary muscle contractions)
	Defecating	Droppings emission
	Brooding	Lying in a nest / on eggs. Including handling eggs with beak
	Self-directed	Tearing feathers, biting wings, legs or ring, etc.
	Repetitive head movements or other abnormal behaviour	Repetitive head movements, repetitive pecking of fences, repeatedly opening wings without flying off, etc.
Directed towards the observer	Observing the observer	Looking at the observer, following them with its eyes. Its attitude is relaxed.
	Vigilant towards the observer	Looking at the observer, following them with its eyes. The posture is tense. Standing or moving
	Threatening	Straightening up, stretching the neck to make itself as tall as possible, high head position, may whistle. Tapping on a hard object (noisy sound) with its beak (may be accompanied by movements of the wings showing the white feathers). Pecks in the direction of the observer
	Running away, hiding	Seeking to move away and hide from the observer
	Seeking the observer's attention	Approaching, seeking attention or contact with the observer (e.g. begging for food)
Other	Not visible	The animal is not easily observable or not visible at all
	Not visible - In the nest	Is not easily observable or not visible at all because it has entered its nest/shelter.
	Aimed at humans	Seeking attention, avoiding or threatening humans in the environment (visitors, staff members)
	Positive towards other species	Positive social behaviours with another species (food sharing, etc.)
	Negative towards other species	Threatening, chasing, pinching, pecking, kicking, etc.
	Undetermined towards other species	Social interaction with another individual that is not on the list or which function has not been identified.
	Unknown / off-list behaviour	Unknown behaviour or which does not correspond to any of the behaviours on the list (e.g.: avoiding an area or an object, shaking oneself).
	Undetermined bill-beating	Pecking against hard, solid objects producing loud sounds. No recipient identified. Excludes threat to observer or negative social behaviour towards conspecific or other species.
	Unknown vocalisation	Vocalisations which function is not known.
