# Supplementary material for: Animal Welfare Monitor: Raising the Bar for Species-Specific Welfare Evaluation Using Welfare Quality® Principles
Source: Animals (Basel). 2026 Mar 7;16(5):842. doi: 10.3390/ani16050842 (PMC12984143; doi:10.3390/ani16050842)
Supplement: Supplementary file 1 [file animals-16-00842-s001.zip › Table_S6_Full_list_AWM_English.pdf]

Table S6: Full list of species covered by the AWM protocol

Class	Order	Family	Species	Total
**Amphibians**				**2**
	**Anurans (2 species)**	Dendrobatidae	Blue poison dart frog (Dendrobates azureus)	1
		Microhylidae	*Tomato frog (Dyscophus)*	1
**Mammals**				**69**
	**Carnivores (13 species)**	Ailuridae	*Red panda (Ailurus fulgens)*	1
		Canidae	*Wolf (Canis lupus), Maned wolf (Chrysocyon brachyurus), Arctic fox (Vulpes lagopus)*	3
		Felidae	*Cheetah (Acinonyx jubatus), Lynx (Lynx lynx), Clouded leopard (Neofelis nebulosa), Lion (Panthera leo), Amur leopard (Panthera pardus orientalis), Amur tiger (Panthera tigris altaica), Snow leopard (Panthera uncia)*	7
		Mustelidae	*Asian small-clawed otter (Aonyx cinereus)*	1
		Ursidae	*Polar bear (Ursus maritimus)*	1
	**Cetartiodactyles (10 species)**	Bovidae	*Domestic goat (Capra aegagrus hircus), Bongo (Tragelaphus eurycerus), Cow (Bos taurus), Takin (Budorcas taxicolor), Sable antelope (Hippotragus niger)*	5
		Camelidae	*Bactrian camel (Camelus bactrianus)*	1
		Cervidae	*Reeves's muntjac (Muntiacus reevesi)*	1
		Girafidae	*Giraffe (Giraffa camelopardalis), Okapi (Okapia johnstoni)*	2
		Suidae	*Domestic pig (Sus domesticus)*	1
	**Diprotodontia (1 species)**	Macropodidae	*Yellow-footed rock-wallaby (Petrogale xanthopus)*	1
	**Perissodactyles (5 species)**	Rhinocerotidae	*White rhinoceros (Ceratotherium simum), Indian rhinoceros (Rhinoceros unicornis)*	2
		Equidae	*Domestic ass (Equus asinus), Grevy zebra (Equus grevyi), Hartmann zebra (Equus zebra hartmannae)*	3
	**Pilosa (1 species)**	Myrmecophagidae	*Giant anteater (Myrmecophaga tridactyla)*	1
	**Primates (38 species)**	Callitrichidae	*Goeldi's monkey (Callimico goeldii), Pygmy marmoset (Cebuella pygmaea), Golden-headed lion tamarin (Leontopithecus chrysomelas), Golden lion tamarin (Leontopithecus rosalia), Silvery marmoset (Mico argentatus), Pied tamarin (Saguinus bicolor), Emperor tamarin (Saguinus imperator), Golden-handed tamarin (Saguinus midas), Cotton-top tamarin (Saguinus oedipus)*	9
		Cebidae	*Brown capuchin (Sapajus apella), Yellow-breasted capuchin (Sapajus xanthosternos)*	2
		Cercopithecidae	*L'Hoest's monkey (Allochrocebus lhoesti), Hamlyn's monkey (Cercopithecus hamlyni), Roloway monkey (Cercopithecus roloway), Eastern black-and-white colobus (Colobus guereza), Celebes crested macaque (Macaca nigra), François' langur (Trachypithecus francoisi)*	6
		Hominidae	*Western lowland gorilla (Gorilla gorilla), Common chimpanzee (Pan troglodytes), Bornean orangutan (Pongo pygmaeus)*	3
		Hylobatidae	*Red-cheeked gibbon (Nomascus gabriellae), Northern white-cheeked gibbon (Nomascus leucogenys), Southern white-cheeked gibbon (Nomascus siki), Siamang (Symphalangus syndactylus)*	4

		Lemuridae	*White-collared lemur (Eulemur cinereiceps), Crowned lemur (Eulemur coronatus), Blue-eyed black lemur (Eulemur flavifrons), Common brown lemur (Eulemur fulvus), Red-bellied lemur (Eulemur rubriventer), Alaotran gentle lemur (Hapalemur alaotrensis), Ring-tailed lemur (Lemur catta), Greater bamboo lemur (Prolemur simus), Crowned sifaka (Propithecus coronatus), Red ruffed lemur (Varecia rubra), Black-and-white ruffed lemur (Varecia variegata)*	11
		Pitheciidae	*Red titi monkey (Callicebus cupreus), White-faced saki (Pithecia pithecia)*	2
	**Proboscidea (1 species)**	Elephantidae	*Asian elephant (Elephas maximus)*	1
	**Rodentia (1 species)**	Hystricidae	*Cape crested porcupine (Hystrix africaeaustralis)*	1
**Bird**				**15**
	**Accipitriformes**	Accipitridae	*Cinereous vulture (Aegypius monachus*)	1
	**Bucerotiformes (2 species)**	Bucerotidae	*Von der Decken's hornbill (Tockus deckeni*)	1
		Bucorvidae	*Northern ground hornbill (Bucorvus abyssinicus*)	2
	**Cariamiformes**	Cariamidae	*Red-legged seriema (Cariama cristata*)	1
	**Casuariiformes**	Casuariidae	*Southern cassowary (Casuarius casuarius*)	1
	**Falconiformes**	Falconidae	*Crested caracara (Caracara plancus*)	1
	**Gruiformes**	Gruidae	*Demoiselle crane (Grus virgo*)	1
	**Psittaciformes (5 species)**	Psittacidae	*Green-cheeked amazon (Amazona viridigenalis*), Hyacinth macaw (*Anodorhynchus hyacinthinus*), Great green macaw (*Ara ambiguus*), Blue-throated macaw (*Ara glaucogularis*), Scarlet macaw (*Ara*	5
	**Sphenisciformes (1species)**	Spheniscidae	*African penguin (Spheniscus demersus*	1
	**Strigiformes (2 species)**	Strigidae	*Little owl (Athene noctua*)	1
		Tytonidae	*Barn owl (Tyto alba*)	1
**Reptile**				**1**
	**Squamate**	Boidae	Madagascar ground boa (Acrantophis madagascariensis)	**1**
			**Total**	87
